# Trips for Health and Pleasure

**Published:** 1906-09-29

**Authors:** 


### TRIPS FOR HEALTH AND PLEASURE.

### III.—The Royal Mail Line to the West Indies.

The invalid or mere pleasure-seeker has now such an extensive selection of routes placed before him that often he must find it no easy matter to decide which will suit him best. Several well-known lines conduct cruises to the Mediterranean, the passenger, as a rule, joining the vessel at Marseilles, which shortens the trip and avoids the much-abused but generally quite harmless "Bay." Many of these cruises are of great interest, but for the invalid they are not sufficiently restful; and the Channel-crossing, followed by a long railway journey, is disliked by many as much as the open sea.

For those who wish to go further afield in their wanderings and who like to visit pastures new, or who are driven out of England by the winter, the Royal Mail Line tours to the West Indies may be freely recommended. The steamers are large and comfortable, and well appointed; and the trips are arranged for various periods so as to suit every requirement of the invalid or tourist. Thus he can join one of the special yachting cruises, which will cost him seventy guineas and give him an eight weeks' holiday; or he can reduce that sum a little by travelling part of the time in one of the ordinary transatlantic steamers. In addition there are eight tours arranged annually for fifty-six days at the fare of £56 and others for seventy days for £70, so that for £1 a day, which includes everything except some of the shore excursions, the voyage may be made with ease, even with luxury. In the fifty-six-days' tour the traveller will return to England by the same steamer as the one he went out by, but of course he must tranship into one of the yachting or intercolonial steamers when he makes the tour of the islands. The stay at Jamaica is from twelve to fifteen days, and hotel expenses there are defrayed by the Company. Passengers are landed and embarked free of charge. It is evident, therefore, that the trips are by no means extravagant.

The Royal Mail steamers leave Southampton on Saturdays, and twelve days afterwards the anchor is dropped off Bridgetown, in Barbados. This island is said to be more homelike than any other West Indian colony, and is sometimes known as "Little England." One of the best social clubs is here, and, under certain conditions, is open to visitors. There are several hotels in Bridgetown or neighbourhood, and *pension* terms range from two guineas a week upwards. The climate of the island is very fine. The average maximum temperature for November, December, January, February, March, and April is 84°, and the average minimum is 70°. The steamer remains at Bridgetown for about six hours, and then moves on to Trinidad, which is reached next morning. Passengers who are making a tour of the islands join the smaller steamer here, and the same evening leave for St. Lucia, the most northerly of the Windward Islands. Dominica is the next stopping-place; and here the day and a half allowed may well be spent in excursions, for the island is, in Mr. Chamberlain's words, "one of the most beautiful and productive in the West Indies." The climate is also fine, and the nights are said to be invariably cool.

Three days and a half after leaving Dominica Jamaica is reached, and the voyagers leave the steamer and stay for about a fortnight at one of the hotels, the ordinary expenses at which are included in the fares. When the fortnight is over the small steamer is again joined, and the *Solent* coasts along Haiti, reaching Porto Rico in about thirty-six hours. In succession he will visit the following places: Martinique,

Grenada, Trinidad, and finally Barbados again. Here the transatlantic steamer is joined and the course made out for Southampton, with occasionally a call at the Azores on the way home. Surely a round like this forms one of the most delightful cruises that can be made in the time and for the amount expended. If the traveller wishes to extend his trip a little he can do so by staying longer at several of the islands, or he may go as far as Demerara, which is easily reached from Trinidad. The temperature at Demerara ranges from 75° to 90° in the shade, but as the north-east trade wind blows from November to April the climate is quite pleasant during these months.

The best season for the invalid to reside in, or the tourist to visit, the West Indies is from October to March; and the former, after looking in at the various islands, ought to be able to make up his mind which will suit him best for a prolonged stay. If he decide on Jamaica, he can move from the sea-level to one of the hill resorts. At Kingston he has the Myrtle Bank Hotel and the Constant Spring, among others; at Moneague the hotel is 1,300 feet about sea-level; at Montpelier, 300 feet; and at Mandeville, 2,000 feet. At Barbados he has a choice of coast hotels such as the Marine and the Crane House. In fact, there is no lack of accommodation, and the charges approximate those of a good English hotel. Nor is there any lack of interest in the surroundings of the invalid. The tropical vegetation, the strange fruits, the scenery, the industries and the costumes of the natives will supply him with material enough for thoughts other than analysing and dwelling on his own health or want of health. As regards outfit, the traveller is advised to take a warm suit for the first and for the last two or three days of the voyage; and for the rest, light, cool clothing is desirable. The time at Jamaica and at Trinidad is long enough to get linen washed; but the tourist is well advised to take sufficient linen with him for the round trip.

